# Supplementary material for: Ratios of Acetaminophen Metabolites Identify New Loci of Pharmacogenetic Relevance in a Genome-Wide Association Study
Source: Metabolites. 2022 May 30;12(6):496. doi: 10.3390/metabo12060496 (PMC9228664; doi:10.3390/metabo12060496)
Supplement: Supplementary file 1 [file metabolites-12-00496-s001.zip › The Qatar Genome Program Research Consortium.pdf]

The Qatar Genome Program Research Consortium

Qatar Genome Project Management: Said I. Ismail[6], Wadha Al-Muftah[6], Radja Badji[6], Hamdi Mbarek[6], Dima Darwish[6], Tasnim Fadl[6], Heba Yasin[6], Maryem Ennaifar[6], Rania Abdellatif[6], Fatima Alkuwari[6], Muhammad Alvi[6], Yasser Al-Sarraj[2,6], Chadi Saad[6] & Asmaa Althani[6,7]

Biobank and Sample Preparation: Eleni Fethnou[7], Fatima Qafoud[7], Eiman Alkhayat[7] & Nahla Afifi[7]

Sequencing and Genotyping group: Sara Tomei[8], Wei Liu[8] & Stephan Lorenz[8]

Applied Bioinformatics Core: Najeeb Syed[9], Hakeem Almabrazi[9], Fazulur Rehaman Vempalli[9] & Ramzi Temanni[9]

Data Management and Computing Infrastructure group: Tariq Abu Saqri[10], Mohammedhusen Khatib[10], Mehshad Hamza[10], Tariq Abu Zaid[10], Ahmed El Khouly[10], Tushar Pathare[10], Shafeeq Poolat[10] & Rashid Al-Ali[10]

Consortium Lead Principal Investigators (in alphabetical order): Omar Albagha[5,19], Souhaila Al-Khodor[11], Mashael Alshafai[12], Ramin Badii[13], Lotfi Chouchane[14], Xavier Estivill[15], Khalid Fakhro[1,4,5,16], Hamdi Mbarek[6], Younes Mokrab[1,4,5,17], Jithesh V. Puthen[5], Karsten Suhre[20,21] & Zohreh Tatari[18]

Affiliations

1 Department of Human Genetics, Sidra Medicine, Doha, Qatar

4 Weill Cornell Medicine-Qatar, Doha, Qatar

5 College of Health and Life Sciences, Hamad Bin Khalifa University, Doha, Qatar

6 Qatar Genome Program, Qatar Foundation Research Development and Innovation, Qatar Foundation, Doha, Qatar.

7 Qatar Biobank for Medical Research, Qatar Foundation, Doha, Qatar.

8 Integrated Genomics Services, Sidra Medicine, Doha, Qatar.

9 Applied Bioinformatics Core, Sidra Medicine, Doha, Qatar.

10 Biomedical Informatics, Sidra Medicine, Doha, Qatar.

11 Microbiome and Biomarkers Discovery lab, Sidra Medicine, Doha, Qatar.

12 College of Health Sciences, Qatar University, Doha, Qatar.

13 Molecular Genetics Lab, Hamad Medical Corporation, Doha, Qatar.

14 Department of Genetic Medicine, Microbiology and Immunology, Weill Cornell Medicine-Qatar, Doha, Qatar.

15 Research Branch, Sidra Medicine, Doha, Qatar.

16 Genomic Medicine Lab, Sidra Medicine, Doha, Qatar.

17 Medical and Population Genomics Lab, Sidra Medicine, Doha, Qatar.

18 Clinical Research Centre, Sidra Medicine, Doha, Qatar.

19 Centre for Genomic and Experimental Medicine, Institute of Genetics and Molecular Medicine, University of Edinburgh, Edinburgh, UK

20 Bioinformatics Core, Weill Cornell Medicine-Qatar, Education City, Doha, Qatar

21 Department of Biophysics and Physiology, Weill Cornell Medicine, New York, NY, USA
